# In Vivo Performance of a Novel Hyper-Crosslinked Carbohydrate Polymer Bone Graft Substitute for Spinal Fusion

**DOI:** 10.3390/bioengineering12030243

**Published:** 2025-02-27

**Authors:** Kee D. Kim, Cynthia A. Batchelder, Plamena Koleva, Arash Ghaffari-Rafi, Tejas Karnati, Dylan Goodrich, Jose Castillo, Charles Lee

**Affiliations:** 1 Department of Neurological Surgery, University of California Davis Medical Center, Sacramento, CA 95816, USA; kdkim@ucdavis.edu (K.D.K.); aghaffarirafi@ucdavis.edu (A.G.-R.); tkarnati@ucdavis.edu (T.K.); djgoodrich@ucdavis.edu (D.G.); jcastillo@ucdavis.edu (J.C.); 2 Molecular Matrix, Inc., 11121 Sun Center Drive Suite C, Rancho Cordova, CA 95670, USA; cabatchelder@molecularmatrix.com (C.A.B.); pmkoleva@molecularmatrix.com (P.K.); 3 Department of Cell Biology and Human Anatomy, University of California, Davis, CA 95616, USA

**Keywords:** bone graft substitute, spinal fusion, arthrodesis, bone fractures

## Abstract

Bone graft materials are essential for achieving arthrodesis after spine surgery. Safe bone graft products, with osteoinductive, osteoconductive properties and the ability to monitor fusion in real-time, are highly desirable. A novel hyper-crosslinked carbohydrate polymer (HCCP) bone graft substitute was shown to aid in bone regeneration in critical-size defect studies in a rabbit model. These studies further evaluated the in vivo application of HCCP as a bone graft substitute in an ovine model of spinal fusion and a retrospective study in adult human spine surgery patients. Sheep studies demonstrated the safety and efficacy of HCCP with no evidence of adverse histopathology over 6 months of follow-up. In human studies, patients (N = 63) underwent posterolateral fusion with HCCP, with follow-up to assess fusion success. No adverse reaction related to the HCCP bone graft substitute was identified. Fusion success was noted to be non-inferior to other bone graft substitutes. HCCP appears to be a safe bone void filler adjunct for use in spinal fusion surgery for both trauma and degenerative disease. It has a good degradation profile for forming bone with the ability to provide new vasculature and may also function as a scaffold to carry cells, medications, and growth factors. Given the safety profile experienced in our preclinical and clinical studies, future investigation into its efficacy to achieve solid fusion is currently ongoing.

## 1. Introduction

Musculoskeletal conditions, encompassing arthritis, injuries, fractures, osteoporosis, back/neck pain, and others, are among the most debilitating nonfatal health conditions worldwide and are the second most common reason patients seek primary care [1]. Bone fractures contribute substantially to disability statistics, with data from the Global Burden of Diseases, Injuries, and Risk Factors Study 2019 demonstrating 178 million new fractures of all causes annually [2]. Spine-related fractures and disorders are the leading cause of disability according to WHO data and are the third most costly health condition in the US [1]. Bone grafting is an important therapeutic tool for addressing fractures and spine issues, with more than 2 million procedures performed annually worldwide [3,4,5,6]. With an aging population, the need for improved bone graft materials and better fracture and spine therapeutic techniques can be expected to increase.

Autologous bone grafts are considered the gold-standard bone repair material as they are osteoinductive and may contain viable precursor cells and growth factors that contribute to osteogenicity [7]. These grafts, taken directly from the patient, carry minimal risk of disease transmission or immune rejection and may be used clinically as cancellous, cortical, vascularized bone, or bone marrow aspirate options. They are collected primarily from the iliac crest but may be derived from proximal tibia, morselized spinous processes, or laminae [8,9]. Collection procedures often require a second surgical site, which extends the time in surgery and increases the risk of potential blood loss, infection, pain, and morbidity for the patient [10,11,12]. In addition, there are limits on the amount of graft material that can be safely harvested from pediatric patients, and there is considerable variation in graft quality due to factors such as the age of the patient, comorbidity and smoking status, and osteoporosis [13,14]. Many of these factors also contribute to delays in bone healing, susceptibility to infection, and post-procedural complications with pain, gait disturbance, numbness, or iliac crest fracture [15,16].

Some of the issues surrounding bone autografts can be mitigated with the use of allogeneic grafts obtained from living or deceased human donors. Allografts are offered in a variety of forms, including, but not limited to, morselized chips, cubes, powders, blocks, sponges, and whole bone segments. Donor bone sources can be fresh, fresh-frozen, freeze-dried, or demineralized bone matrix (DBM) yielding a range of handling characteristics and osteoconductive properties. In contrast to bone autografts, allografts are available in commercially prepared, off-the-shelf products providing efficient, ready-to-use biocompatible bone filler material [17]. Variability in donor bone, age, sterilization, and processing techniques have significant consequences on allograft biological and mechanical properties; thus, lot-to-lot comparisons and controlled studies are challenging. Even with strict donor serological disease testing, some risk of disease transmission remains along with potential host immune response or rejection, issues with low mechanical strength, and prolonged healing time [18,19].

Synthetic grafts provide an attractive alternative for bone repair or reconstruction as they are more readily available and can be found as commercialized, off-the-shelf formulations. Polysaccharides such as alginate and chitosan have been investigated previously for their role in bone repair and regeneration for several decades [20,21,22,23]. A novel carbohydrate polymer (HCCP) was generated by hyper-crosslinking polysaccharide chains of glucose and mannose and previously investigated in vitro and in vivo [24,25,26]. HCCP shows clinical promise for the treatment of bone defects without the risk of immunogenic complications or adverse fibrotic tissue responses. In further studies, HCCP was investigated for the repair of critical-sized bone defects in comparison to two alternative treatments, autologous bone and poly(lactide-co-glycolide) with hyaluronic acid (PLGA/HA). Bilateral critical-sized defects were created in the lateral femoral condyles of skeletally mature rabbits and subsequently implanted with HCCP, PLGA/HA, or autologous bone in a randomized manner [26]. Defects implanted with HCCP showed progressive bone regeneration and bridging of the defect without adverse histological events or signs of infection or inflammation. At the 16-week radiographic assessment, bone density and volume were significantly higher in defects implanted with HCCP compared to PLGA/HA. Histologically, significant degradation of HCCP was noted by 4 months post-implant. No statistically significant difference was observed in bone density and volume between HCCP and autologous bone.

Large animal models are an essential translational step prior to clinical studies in human patients, as they more closely reflect the anatomy, physiology, and mechanical loading of the human spine [27,28,29,30,31]. The goals of this study were to (i) evaluate the safety and efficacy of HCCP bone graft substitute for interbody spinal fusion in an ovine preclinical model and (ii) conduct a retrospective review to evaluate the single-center safety and clinical application of HCCP as an adjunct bone graft substitute.

## 2. Materials and Methods

### 2.1. Pre-Clinical Studies, Animal Care and Use

The Sonoma County Institutional Animal Care and Use Committee (SCIACUC # SCACU0114) approved all procedures involving live animals. Twelve mature male crossbred Suffolk/Dorset sheep (age 18–36 months) were used. The animals were randomly distributed into 2 groups (N = 6 per group): HCCP (Osteo-P^®^ Bone Graft Substitute, Molecular Matrix, Inc., Carlsbad, CA, USA) and autograft. All animals were acquired from a single commercial source and housed for a 28-day acclimation period. All animals were fed pellets consisting of corn, alfalfa, and hay twice daily throughout the study. On the day of the procedure, animals were administered Ketamine (15 mg/kg) IV for sedation. Lidocaine (1%) was applied on the skin and subcutaneous tissue, then directly to the underlying periosteum to anesthetize the aspiration site. All animals were fasted for 12–24 h before surgery.

### 2.2. Surgical Procedure, Pre-Clinical Study

With the animal in right lateral recumbency and in Trendelenburg position to accommodate draining of rumen and saliva, the wool was removed from the left lateral lumbar area and the skin prepared for aseptic surgery. Anatomical palpation and fluoroscopy were used to identify the disc spaces between L4 and L5 with a lateral retroperitoneal approach. Discectomy was performed, and endplates were prepared to accept the vertebral spacer with standard disc prep instruments. The annulus on the contralateral side was left intact to prevent lateral expulsion of the cage. Before insertion of the lateral interbody cage, the disc space was prepared, and a trial implant was used to ensure complete approximation of the endplates with an appropriate interbody cage size. A surgical scalpel was used to cut the HCCP implant to the size of the inner dimensions of the spinal cage. If needed, a vertebral spreader was used to open the disc space. The cage plus bone graft substitute (HCCP or autograft) was inserted into the intervertebral space using standard insertion instruments. Care was taken to ensure consistent vertebral body distraction, and impaction force was used in all animals. The surgical site was irrigated with an antibiotic solution consisting of sterile saline and Cephazolin sodium. Routine closure of external muscular fascia, subcutaneous tissue, and skin was performed. Animals were administered perioperative antibiotics (Cephazolin), and pain management procedures were employed.

### 2.3. Pre-Clinical Radiography and Histology

The fusion status was assessed by computed tomography (CT) six months after surgery. After the animals were sacrificed the spinal segments were dissected in the sagittal plane and collected in 10% formalin with decalcification solution. Decalcified sections were embedded, sectioned, and stained with hematoxylin and eosin. Histopathological analysis was performed under blinded conditions.

### 2.4. Clinical Study Ethics Approval

All aspects of this research endeavor were conducted in compliance with the Declaration of Helsinki, Good Clinical Practice (GCP) guidelines, and applicable local and international regulations. The Institutional Review Board of the University of California, Davis, approved a waiver of patient authorization to use/disclose PHI following a determination that the use or disclosure of the protected health information involved was minimal risk to the privacy of individuals (IRB# 1969904-1).

### 2.5. Clinical Study Design

A case series of patients who underwent a posterolateral, cervical, thoracic, and/or lumbar instrumented arthrodesis with HCCP bone graft substitute was retrospectively reviewed. All cases performed from April 2019 to April 2020 at the University of California, Davis Medical Center were reviewed. Table 1 summarizes demographic and clinical characteristics of the patient cohort. HCCP was used at the attending discretion at the time of surgery for either degenerative or trauma spine surgeries.

### 2.6. Clinical Study Objectives

The primary objective of the study was to investigate the safety and efficacy of the spinal fusion procedure in patients receiving HCCP bone graft substitute. Potential complications and long-term fusion outcomes were noted.

### 2.7. Clinical Surgical Technique

In all cases, HCCP was used as an adjunctive graft product for arthrodesis after posterior instrumented fusions. Surgical technique for instrumentation, number of levels treated, and use of other graft products was carried out based on the clinical judgment of the attending spine surgeon. HCCP was used with some combination of autograft, allograft, and/or demineralized bone matrix (DBM) for arthrodesis of involved segments over the posterior or posterolateral elements that had been prepared for fusion. HCCP was provided as a sheet to apply to the implant site without any further material preparation. At the discretion of the attending surgeon, growth factors such as bone morphogenetic proteins (BMP) or commercially available combination devices such as Infuse^®^ Bone Graft (Medtronic, Minneapolis, MN, USA) were used.

### 2.8. Clinical Data Collection

A comprehensive review of electronic health records was conducted to assess the surgical aspects of the procedure, including the specific type of surgical intervention, the involved spinal levels, instrumentation utilized, and any bone graft substitutes or adjuncts employed. All patient data was collected, then de-identified, in compliance with privacy and confidentiality regulations to protect patient information. Radiographs and radiology reports were reviewed for follow-up visits to assess spinal fusion. Charts were reviewed for any post-operative complications, including those possibly attributable to use of HCCP. When available, follow-up imaging was reviewed to evaluate any evidence of early hardware failure or pseudoarthrosis.

### 2.9. Data Analysis and Statistics

Data were analyzed using appropriate statistical methods, including descriptive statistics such as mean, standard deviation, and frequency (proportion) of all patients, where applicable. The effects of patient age, comorbidities, or graft biomaterials on outcomes were analyzed by Student’s *t*-test or ANOVA. Statistical significance was considered at *p* < 0.05.

## 3. Results

### 3.1. Pre-Clinical Safety and Efficacy of HCCP

The safety and efficacy of HCCP were evaluated in an ovine model of interbody spinal fusion. No significant safety issues were noted with the use of graft materials. Animals were studied under CT scan after removing instrumentation.

**Figure 1:**
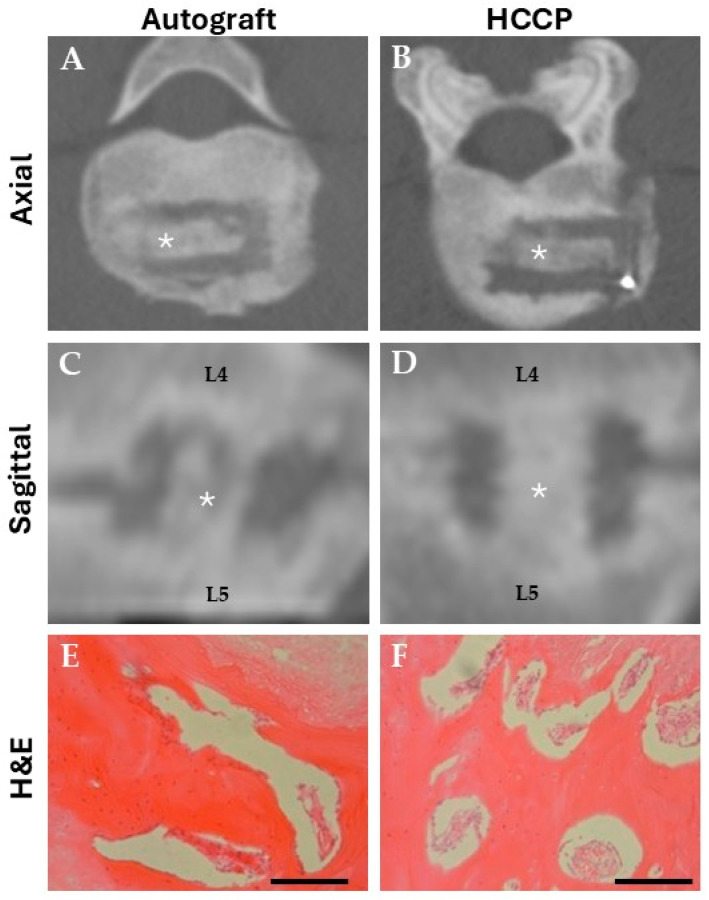
**Evaluation of spinal fusion in sheep by computed tomography (CT) and histopathology, 6 months postoperatively.** Bone growth as determined by CT was equivalent in autograft (**A**,**C**) and HCCP (**B**,**D**) treatment groups; graft location is denoted by an asterisk (*). Region of fusion: Lumbar 4 (L4) to Lumbar 5 (L5). No abnormalities were noted in Hematoxylin and Eosin (H&E) stained sections, autograft (**E**), and HCCP (**F**). Scale bars = 500 µm.

An axial image reconstructed from all studied levels showed new bone covering the vertebral bodies, with a similar amount of bone observed in the autologous and HCCP groups at the 6-month follow-up (Figure 1A–D). Spinal segments were collected at tissue harvest and assessed for histopathology. No signs of histologic abnormalities were noted (Figure 1E,F).

### 3.2. Clinical Study Demographics and Comorbidities

Retrospective clinical study demographics are provided in Table 1. There were 23 females and 40 males with a mean age of 65.0 ± 14.7 years (range 23–96 years) in the patient cohort. Comorbidities included diabetes (22.2%), smoking (current or former, 55.5%), osteoporosis (12.9%), hypertension (54.0%), hyperlipidemia (28.6%), or long-term steroid use (4.7%). The mean body mass index (BMI) was 28.8 ± 6.5 kg/m^2^. Neither the age of the patient nor the comorbid condition affected fusion outcomes (*p* > 0.05).

### 3.3. Clinical Study Fusion Outcome by Spinal Level

Fusion rates were assessed by spinal region (Table 2). Of the total fusion procedures, 23 were cervical, 12 were thoracic, and 26 were lumbar (comprising 36.5, 19.0, and 41.3% of total procedures, respectively). The number of segments fused was 5.25 cervical (SD: 2.8; range 1–10), 4.5 thoracic (SD: 2.6; range 1–7), and 4.1 lumbar (SD: 3.8; range 1–12) with no significance detected between regions, *p* > 0.05. Overall, the fusion rate with HCCP utilized as an adjunct bone graft substitute was 91.8%. Fusion rates were compared between cervical (85.0%), thoracic (100.0%), and lumbar regions (95.0%) with no significant effect of region noted, *p* > 0.05.

### 3.4. Clinical Application of Bone Graft Adjunct Materials, Instrumentation, and HCCP

HCCP was used as a bone graft substitute at the attending spine surgeon’s discretion, and the combination of products used reflects current therapeutic recommendations. Spinal fusion was successful in patients receiving HCCP in combination with autograft, allograft, demineralized bone matrix (DBM), or combinations thereof and with/without bone morphogenetic protein-2 (BMP-2) (Table 3).

At the attending surgeon’s discretion, some patients received a combination of cages, screws, and/or rods in combination with HCCP and other bone graft adjuncts. HCCP is highly porous and can be shaped and molded to fit a variety of clinical applications, as demonstrated by the number of combinations in which it was used by clinicians. No safety or performance issues were observed related to HCCP addition as a bone graft substitute, demonstrating the versatility of this material in a variety of clinical applications.

### 3.5. Safety and Efficacy of HCCP in Human Patients

**Figure 2:**
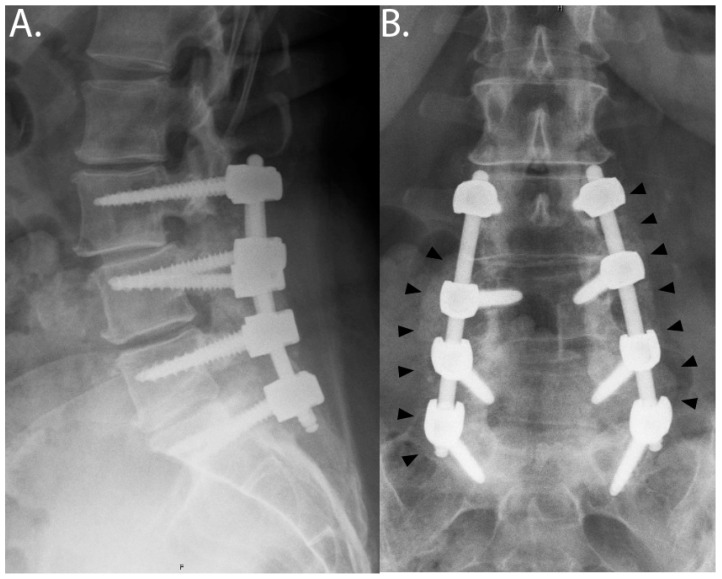
**L3-S1 posterior instrumented fusion utilizing HCCP.** Bony fusion is annotated with small black arrows. Upright x-rays were taken 13 months post-operatively: (**A**) lateral, (**B**) anterior-posterior views.

HCCP was well-tolerated in patients. No adverse events related to the use of HCCP as an adjunct bone graft substitute were observed. Bone repair was assessed for fusion radiographically. Regions fused with HCCP demonstrated excellent bone repair with visible osseous formation within 3 months postoperatively and comparable efficacy with autograft or allograft alone at 6 months postoperatively. HCCP was observed to be radiolucent, allowing new bone formation to be visualized in real-time by radiograph or CT, without the noted interference from other bone graft substitutes, which may be radiopaque or mineralized. Representative images of lumbar fusion are shown in Figure 2.

## 4. Discussion

Bone graft materials are essential for achieving arthrodesis after spine surgery. Safe bone graft products, with properties that are osteoinductive, osteoconductive, and with the ability to monitor fusion in real-time, are highly desirable [32,33,34,35]. HCCP bone graft substitute is a novel hyper-crosslinked carbohydrate polymer shown to aid in bone regeneration both in vitro and in vivo preclinical studies [26]. We report the safety and efficacy of HCCP bone graft substitute in an ovine model of spinal fusion and the translation of these results to human patients via a retrospective clinical study. Further studies are ongoing to elucidate the mechanism of bone regeneration by HCCP, including exploration of growth factor retention, potential signaling pathways, establishment of new vasculature, and the migration, proliferation, and differentiation of mesenchymal stem cells into the highly porous microstructures of HCCP.

Small ruminants such as sheep and goats are among the most common preclinical models for spinal fusion studies. Sheep are the most widely used small ruminants for such studies due to availability, ease of handling, size, and other practical and financial considerations [27,29]. At skeletal maturity, ovine vertebral structure and function is highly analogous to the human spine, and the ovine model is commonly used for assessment of implant efficacy [28,30]. Importantly, no safety issues were observed, and the HCCP implant was as effective as autologous bone in this model, lending support for FDA clearance of this material as a bone void filler.

Spinal arthrodesis, or fusion, is frequently recommended for pathological conditions occurring in the cervical, thoracic, and lumbar spine as well as surgical issues involving trauma, bone deformity, and degeneration [32]. The global health burden of obesity, along with the aging population and the concomitant increase in osteoporosis, points to a continual increase in the demand for synthetic bone graft substitutes [33,34,35,36,37]. Carbohydrate-based polymers/scaffolds may be used as bone graft substitutes for bone regeneration owing to their structural similarities with glycosaminoglycans (GAGs); consequently, they may mimic the function of GAGs (bone repair and regeneration) in vivo with a favorable resorption-to-regeneration rate profile [26]. GAGs upregulate the genes involved in osteogenesis and promote osteoblastic differentiation of mesenchymal stem cells (MSCs). Prior preclinical studies demonstrated HCCP to be biocompatible, osteoconductive, and capable of promoting bone regeneration in vivo. Furthermore, the carbohydrate polymer nature of HCCP did not elicit an immune response or adverse inflammatory response, nor did it contribute to any systemic toxicity post-implantation in previous animal studies [26]. Results of this study demonstrate similar properties of HCCP in ovine models and in human clinical applications for spinal fusion.

Synthetic grafts provide an attractive alternative for bone repair or reconstruction as they are more readily available and can be found as commercialized, off-the-shelf formulations. This class of bone graft substitutes includes materials that are inert, non-toxic, free of disease transmission risk, non-immunogenic, and produced more economically than other graft materials [38]. While lacking the osteogenic and osteoinductive properties of autologous grafts, synthetics may be osteoconductive with similarities in mineral composition to native bone and have the potential to stimulate differentiation and activity of osteoblasts [38,39]. The physical and chemical characteristics of each synthetic contribute important properties for bone repair: varying pore sizes, porosity, brittleness, rate of resorption, and mechanical properties such as tensile strength or compressive modulus [39]. Calcium sulfate is attractive due to its ease of use and low cost but is limited to small void filling and non-weight-bearing applications due to rapid resorption [40]. Ceramics such as hydroxyapatite (HA), β-tricalcium phosphate (β-TCP), or calcium phosphate are available in a variety of powders, pastes, and glues but are limited by incomplete or variable resorption rates and the formation of immature fibrous tissue, which frequently causes incomplete integration and a lack of sufficient load-bearing strength [30,41]. They are typically utilized as bone graft substitutes or extenders to augment instrumented fusion. Synthetic polymers such as poly(lactic-co-glycolic acid) (PLGA) or polymethylmethacrylate (PMMA) have excellent biocompatibility and more controlled degradation rates, but wettability properties may limit protein or cell attachment unless modified [38]. Bioactive glasses are a group of silica-derived polymers that form rapid, strong bonds with bone and soft tissues and appear to stimulate osteoblast activity but are limited by mechanical reliability and low resistance to fracture in weight-bearing applications [42].

The clinical study described here is a retrospective analysis of all the patients who received HCCP as an adjunct bone graft substitute over a 12-month period. There are inherent statistical limitations to retrospective studies with a limited number of patients and results may not fully extrapolate to other patients. Here, the patient population was predominantly male (63.5%), which may have influenced outcomes in a positive direction. While biological sex alone is not expected to influence spinal fusion outcomes, female sex has been a predictive factor for negative outcomes regarding pain and disability, though not necessarily clinical outcomes [43,44,45]. The inclusion of older females, a cohort more likely to be affected by osteoporosis as a comorbidity, may influence outcomes positively or negatively depending on treatment status [46,47]. A larger study with a significantly higher number of subjects is currently being conducted to provide statistically significant insights and trends.

As a carbohydrate polymer, HCCP combines many of the desirable characteristics of synthetic bone graft substitutes: ease of use, moldability, versatility, porosity, and the ability to support cell attachment and remodeling. Further, HCCP may undergo chemical modification to improve osteoinductive and osteogenic characteristics. Fusion success in the studies reported here was noted to be non-inferior to other bone graft substitutes. HCCP appears to be a safe adjunct graft product for use in spinal fusion surgery for both trauma and degenerative disease. As an off-the-shelf product with a favorable degradation to bone regeneration profile, HCCP offers a non-immunogenic alternative to improve bone fusion surgery outcomes. Previous in vivo studies in rabbit femoral condyle models demonstrated the ability to support new vasculature [26]. The biochemical and physical nature of HCCP may also function as a scaffold to carry cells, medications, and growth factors. Given the safety profile experienced in our preclinical and clinical studies, future investigation into its efficacy to achieve solid bony fusion and to improve bone fracture repair is currently ongoing.

## 5. Conclusions

In this retrospective study, the clinical applicability and safety of HCCP as a bone graft substitute were demonstrated in an ovine model of spinal fusion and in adult human spine surgery patients. No adverse reactions related to HCCP were identified. Fusion success was noted to be non-inferior to other bone graft substitutes. HCCP appears to be a safe adjunct graft product for use in spinal fusion surgery for both trauma and degenerative disease. It has a good degradation profile for forming bone with the ability to provide new vasculature and may also function as a scaffold to carry cells, medications, and growth factors. Given the safety profile observed in these preclinical and clinical studies, future investigation into its efficacy to achieve solid fusion is currently ongoing.

## Figures and Tables

**Table 1:** Clinical Demographic Information and Comorbidities (N = 63).

Age (yr)	N	(%)	SD	Smoking	N	(%)
<40	4	(6.4)		Yes	35	(55.5)
41–49	6	(9.5)		No	28	(44.5)
50–59	10	(15.9)		Former > 10 yr	8	
60–69	16	(25.4)		Former ≤ 10 yr	6	
70–79	19	(30.2)		Former (unspecified)	3	
80–89	7	(11.1)				
90+	1	(1.6)				
**Mean Age (yr)**	**65.0**		**14.7**			
**Gender**				**Osteoporosis**		
Female	23	(36.5)		Yes	8	(12.9)
Male	40	(63.5)		No	13	(21.0)
				NA	41	(66.1)
**Diabetes**						
Yes	14	(22.2)		**Steroid Use**		
No	48	(76.2)		Yes	3	(4.7)
NA	1	(1.6)		No	60	(95.3)
**BMI (kg/m^2^)**	**28.8**		**6.5**			
**Hypertension**				**Hyperlipidemia**		
Yes	34	(54.0)		Yes	18	(28.6)
No	26	(41.3)		No	42	(66.7)
NA	3	(4.7)		NA	3	(4.7)

**Table 2:** Spinal Fusion Procedures with HCCP by Region.

Region	Number of Procedures	Fused *n*, (%)	Not Fused *n*, (%)	Lost to Follow-Up *n*, (%)
Cervical	23	17, (85.0)	3, (13.0)	3, (13.0)
Thoracic	12	8, (100.0)	0	4, (33.3)
Lumbar	26	19, (95.0)	1, (3.8)	6, (23.1)
Unspecified	2	1, (100.0)	0	1, (50.0)
All Levels	63	45, (91.8)	4, (6.3)	14, (22.2)

**Table 3:** Clinical Study Fusion Rate by Bone Graft Material.

Bone Graft Adjunct Material	Number of Procedures	Fused (N)	Not Fused (N)	Lost to Follow-Up * (N)	Fusion ** (%)
Autograft + Allograft + HCCP	19	13	1	5	13/14 (92.9)
Autograft + Allograft + DBM + HCCP	10	7	2	1	7/9 (77.8)
Autograft + HCCP	13	8	0	5	8/8 (100.0)
Allograft + HCCP	5	5	0	0	5/5 (100.0)
Autograft + Allograft + BMP + HCCP	5	5	0	0	5/5 (100.0)
Allograft + DBM + HCCP	3	1	0	2	1/1 (100.0)
Autograft + DBM + HCCP	3	2	1	0	2/3 (66.7)
Allograft + BMP + HCCP	2	2	0	0	2/2 (100.0)
Autograft + Allograft + InFuse + HCCP	1	1	0	0	1/1 (100.0)
HCCP	2	1	0	1	1/1 (100.0)
**Total**	**63**	**45**	**4**	**14**	**45/49 (91.8)**

* Lost to follow-up is defined as those patients who failed to return for evaluation prior to 4 months post-surgery. ** Number of patients with radiographic fusion divided by the number of patients who completed follow-up. Abbreviations: HCCP: hyper-crosslinked carbohydrate polymer, DBM: demineralized bone matrix, BMP: bone morphogenetic protein-2.

## Data Availability

Data presented in this study are available on request from the corresponding author.
